# Mitochondrial Remodeling, Cytoskeletal Dynamics, and Translational Control During *Drosophila* Spermatid Elongation: An Integrative Review

**DOI:** 10.3390/biology15171562

**Published:** 2026-09-07

**Authors:** Yang Fang, Jiaxin Yin, Liping Chen, Fengchao Zhang, Yang Liu, Liang Ma, Xiwen Liu, Yang Luo, Hua He, Qijun Xie

**Affiliations:** 1 Hunan Key Laboratory of Biomedical Nanomaterials and Devices, School of Biological Science and Medical Engineering, Hunan University of Technology, Zhuzhou 412007, China; fangyang@hut.edu.cn (Y.F.); 23408270221@stu.hut.edu.cn (J.Y.); 2507100202010@stu.hut.edu.cn (L.C.); claralin527@hut.edu.cn (L.M.); liuxiwen@hut.edu.cn (X.L.); luoyang@hut.edu.cn (Y.L.); 2 College of Marine Life Sciences, Ocean University of China, Qingdao 266003, China; zhangfengchao2214@stu.ouc.edu.cn (F.Z.); liuyang9573@stu.ouc.edu.cn (Y.L.); 3 Laboratory Medicine Center, Zhuzhou Hospital Affiliated to Xiangya School of Medicine, Central South University, Zhuzhou 412007, China

**Keywords:** *Drosophila melanogaster*, spermatid elongation, mitochondrial remodeling, post-transcriptional regulation, individualization, cytoskeletal dynamics

## Abstract

Spermatogenesis in *Drosophila melanogaster* is a classical model for studying cell polarity establishment, organelle remodeling, and extreme cellular morphogenesis. Spermatid elongation involves a series of highly coordinated dynamic events, including nuclear polarization, axonemal extension, mitochondrial remodeling, and individualization-mediated cytoplasmic clearance. Current studies have largely focused on individual structures, molecules, or pathways, resulting in a fragmented understanding of this process and a lack of an integrated framework that links mitochondrial function, cytoskeletal dynamics, and temporal translational regulation. Based on existing evidence, this review organizes current findings into three regulatory layers involving mitochondrial remodeling, cytoskeletal dynamics, and post-transcriptional translational control. Mitochondrial remodeling provides structural and metabolic support for sperm-tail formation, cytoskeletal processes execute axoneme organization and individualization, and post-transcriptional regulation controls the timing of protein production. Experimental evidence is strongest for mechanisms operating within each layer, whereas direct molecular coupling among the three layers remains incompletely established. We therefore present this model as a working framework rather than an established causal pathway. This framework helps organize current knowledge of extreme cellular differentiation and provides a basis for comparative studies of spermatogenesis and selected mechanisms associated with human male infertility.

## Simple Summary

Spermatogenesis in *Drosophila melanogaster* provides a powerful model for understanding how a round germ cell is transformed into a highly elongated and functional sperm cell. Previous studies have identified many factors involved in sperm tail elongation and individualization, but these processes are often discussed separately. In this review, we bring these observations together and organize them into three regulatory layers: mitochondrial remodeling, cytoskeletal dynamics, and post-transcriptional translational control. Mitochondria not only contribute to energy production, but also to sperm tail structure; the cytoskeleton provides the scaffold and mechanical force needed for elongation and individualization; and delayed translation helps ensure that key proteins are produced at the correct developmental stage. This integrated view, while recognizing that direct connections among the three layers remain incompletely established, may help us to clarify how extreme cell morphologies are generated and provide useful reference points for understanding selected mechanisms of human male infertility.

## 1. Introduction

**Figure 1:**
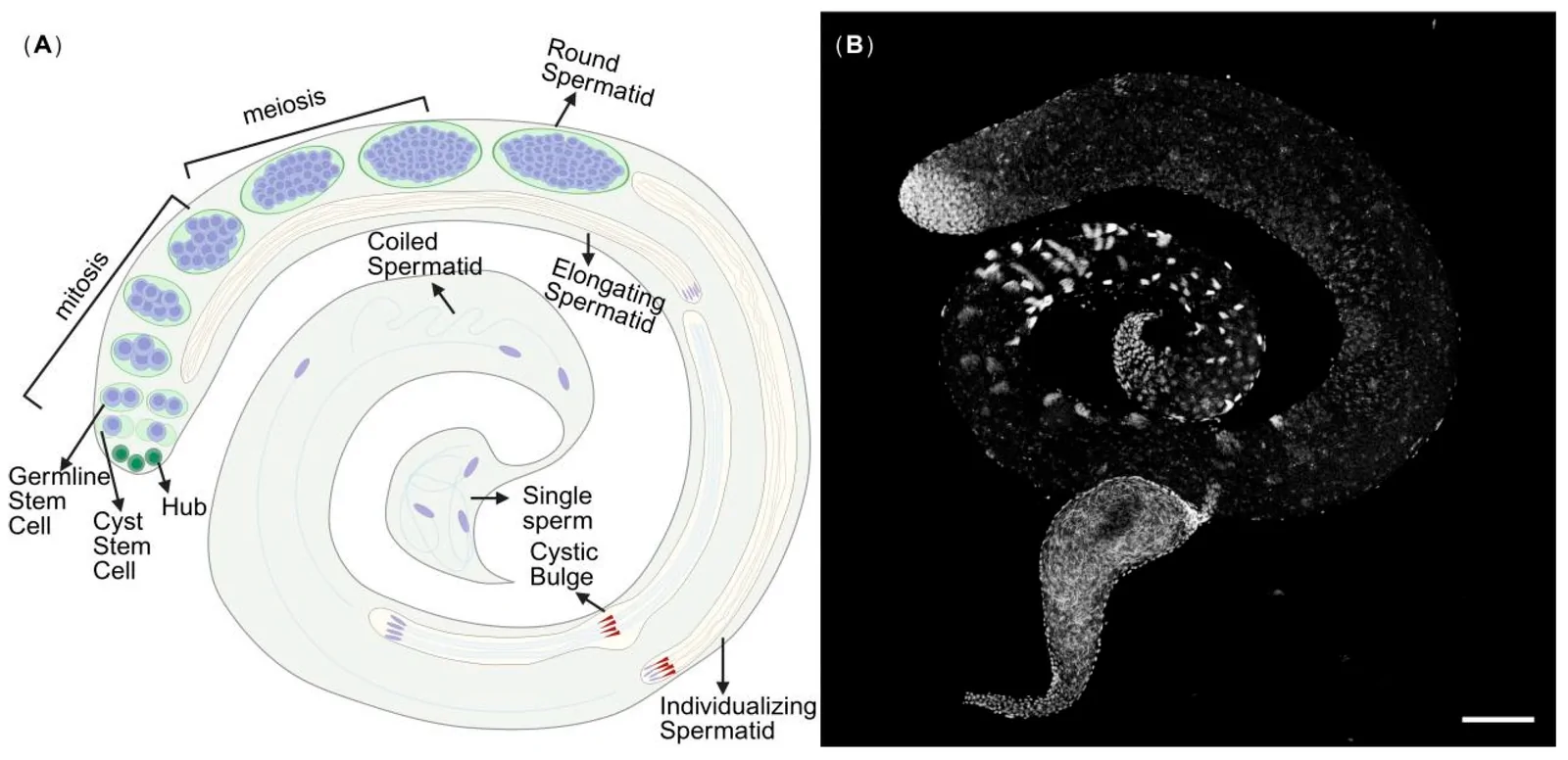
Schematic overview of *Drosophila* spermatogenesis and whole-testis DAPI staining. (**A**) Schematic illustration of the continuous developmental progression in the *Drosophila* testis, from germline stem cells, spermatogonia, spermatocytes, round spermatids, and elongating spermatids to mature sperm. (**B**) Original whole-testis DAPI staining image of an adult male *Drosophila* testis acquired by the authors, showing the spatial distribution of nuclei at different developmental stages within the testis. DAPI labels nuclear DNA. This figure indicates the position of spermatid elongation, the main focus of this review, within the overall process of spermatogenesis. The microscopy image shown in (**B**) is original and was generated by the authors; it was not reproduced or adapted from a previously published source. Scale bar: 100 μm.

*Drosophila melanogaster* provides a well-established system for dissecting the developmental events of spermatogenesis [1]. Germ-cell development starts at the apical testis niche, where GSCs divide asymmetrically to generate gonialblasts. Four synchronous mitotic divisions then expand each gonialblast into a 16-cell spermatogonial cyst. The daughter cells remain linked through cytoplasmic bridges and develop within an enclosure formed by cyst cells [2,3]. These spermatogonia next grow into primary spermatocytes and undergo meiosis I and II, yielding 64 haploid round spermatids per cyst. The cells subsequently proceed into spermiogenesis. First, the basal body nucleates the extension of the microtubule-based axoneme, while cytoplasmic microtubules and F-actin filaments are reorganized along the longitudinal axis to establish cell polarity [4,5,6]. Second, the spermatid nucleus undergoes extensive chromatin condensation and elongates into a needle-like structure, while the mitochondrial derivatives extend synchronously along the axoneme [7]. Finally, the individualization complex migrates from the head region toward the tail, removing excess cytoplasm and organelles, which are collected and degraded in the terminal waste bag (Figure 1A). Through this process, each sperm cell ultimately acquires an independent and functional mature morphology [8,9,10] (Figure 1A).

Current studies on *Drosophila* spermatid elongation have largely focused on specific structures or individual regulatory molecules, and an integrated systematic framework has not yet been fully established. Existing work has examined several key processes, including cytoskeletal connections involved in nuclear elongation, the formation and maintenance of mitochondrial derivatives, and the assembly and migration of the individualization complex [11,12,13]. These studies have provided important mechanistic insights into specific aspects of spermatid elongation. However, the intrinsic relationships among these findings, the temporal order of different regulatory events, and the logic underlying their coordination remain insufficiently understood. Although the major events of spermatid elongation have been well described, how these events are integrated into a coordinated biological process remains a central question in the field.

To understand the regulatory mechanisms underlying spermatid elongation, it is necessary to first summarize the major research directions in which substantial progress has been made. Current studies have mainly focused on three aspects. The first is mitochondrial regulation [14,15]. Proper formation and elongation of mitochondrial derivatives provide both metabolic support and structural stability for spermatid elongation. Several genes that affect mitochondrial morphology and function have been identified, including *ND-42*, which is involved in regulating mitochondrial membrane potential, and *Vha68-3*, which participates in mitochondrial metabolic remodeling [12,14]. Additional cilia-related genes have also been shown to influence mitochondrial dynamics during spermatogenesis [16]. The second aspect is translational regulation [17,18,19]. During late spermatogenesis, transcriptional activity is markedly reduced as chromatin progressively condenses, and gene expression therefore largely depends on the selective translation of pre-synthesized mRNAs. The third aspect is cytoskeletal dynamics [20,21,22]. Microtubules and F-actin constitute the structural framework required for spermatid elongation. During axoneme assembly, multiple microtubule-associated proteins participate in dynein arm anchoring and axonemal stabilization. During migration of the individualization complex, several actin-binding proteins regulate its directional movement along the spermatid tail. In addition, proteins that connect the nuclear pore complex with microtubule bundles have also been shown to be required for nuclear elongation [23].

Although mitochondrial regulation, translational timing, and cytoskeletal remodeling have each been extensively studied during *Drosophila* spermatid elongation, these processes are still often examined separately. Much of the available evidence comes from analyses linking individual genes to specific developmental defects, leaving the relationships between different cellular events less well defined. In particular, it remains unclear whether changes in mitochondrial metabolism directly affect cytoskeletal organization, how the timing of protein synthesis is matched to the demands of elongating spermatids, and what mechanisms synchronize nuclear shaping, mitochondrial derivative elongation, and individualization. In addition, whether upstream nutrient-sensing pathways, such as TOR signaling, contribute to the coordination of these three regulatory layers remains unclear. More importantly, these dispersed findings have yet to be incorporated into an integrative explanatory framework that connects structural remodeling, energy metabolism, and post-transcriptional regulation.

To address these issues, this review proposes a multilayered regulatory model for *Drosophila* spermatid elongation. During spermiogenesis, axoneme extension, nuclear elongation, mitochondrial derivative formation, and actin-based individualization occur in a highly ordered developmental sequence (Figure 1A), providing the structural and temporal context for examining their potential coordination. The findings discussed above can be grouped into three closely related aspects: mitochondrial metabolism, translational regulation, and cytoskeletal dynamics. Considering these processes together may help explain how structural remodeling, metabolic support, and stage-specific protein production are coordinated during *Drosophila* spermatid elongation. Importantly, the three layers are not supported by equivalent levels of mechanistic evidence: whereas their individual roles in spermatid development are relatively well established, several proposed cross-layer interactions remain inferred from genetic, morphological, or temporal associations and require direct experimental validation. Given that *Drosophila* and mammalian spermatogenesis share conserved features in axoneme formation, chromatin condensation, mitochondrial function, and post-transcriptional regulation, the *Drosophila* model may provide useful insights into cellular processes associated with male infertility, particularly when spermatid elongation is considered within the full developmental sequence from germline stem cells to mature sperm (Figure 1).

## 2. Structural Framework of Spermatid Elongation

The apical region of the *Drosophila* testis contains both germline stem cells (GSCs) and cyst stem cells (CySCs), which together initiate formation of the developing germline cyst. Each GSC division retains one stem cell and produces a gonialblast, while CySC progeny differentiate into the cyst cells that surround the germline [24,25]. The gonialblast then undergoes four incomplete and synchronous mitotic divisions, yielding a 16-cell spermatogonial cyst whose cells remain linked by cytoplasmic bridges. These spermatogonia subsequently enlarge and differentiate into primary spermatocytes. Throughout this process, cyst cells derived from cyst stem cells extend their plasma membranes to fully enclose the germ cell clone within a sealed cystic compartment. Under the enclosure of cyst cells, spermatocytes complete meiosis and ultimately generate a syncytial cyst composed of 64 haploid round spermatids [26,27].

As spermatids begin to elongate, the cyst acquires a clear head-to-tail polarity. The two somatic cyst cells adopt different positions and morphologies: the smaller head cyst cell remains associated with the clustered spermatid nuclei, whereas the larger tail cyst cell occupies the opposite end of the growing bundle. Consequently, the 64 nuclei are grouped at one end of the cyst, while the developing tails extend in parallel toward the tail cyst cell. The spermatids nevertheless remain connected through cytoplasmic bridges during this extensive remodeling, allowing the cyst to retain its syncytial organization and facilitating the shared distribution of developmental and metabolic components [28,29]. This polarized arrangement defines the orientation of the sperm bundle and provides the spatial context for the axoneme extension, nuclear positioning, and subsequent remodeling events described below.

After cyst polarity is established, spermatids enter a phase of extensive elongation, during which the intracellular cytoskeleton and membrane systems undergo large-scale remodeling and spatial reorganization. The axoneme, which serves as the main structural scaffold of the sperm tail, originates from the basal body and extends continuously toward the distal end of the cyst. In cross-section, the axoneme displays the conserved “9+2” microtubule arrangement, in which nine peripheral doublet microtubules surround a central pair of singlet microtubules. Inner and outer dynein arms assembled on the peripheral doublets provide the structural basis for subsequent sperm motility [30,31,32,33,34,35]. As the axoneme extends distally, the nucleus, originally located near the central region of the cell, undergoes chromatin condensation and dramatic volume reduction, gradually transforming from a round structure into a compact needle-like shape. The nucleus then becomes directionally positioned in association with the elongation axis, and all nuclei are ultimately anchored at the head cyst cell side. Meanwhile, the plasma membrane expands in coordination with the extension of the internal cytoskeletal framework. A submembranous actin network provides mechanical support for the cell during this extensive morphological remodeling. At the late elongation stage, the membrane-associated actin structures further organize into the individualization complex, which contains actin bundles and migrates along the sperm bundle from head to tail. This complex progressively removes excess cytoplasm and ultimately separates the 64 interconnected spermatids into individual mature sperm.

Previous studies have established several major structural and cellular requirements for *Drosophila* spermatid elongation, including cyst-cell organization [24,25,26,27,28,29], axoneme assembly [30,31,32,33,34,35], nuclear shaping [7,23], and actin-based individualization [8,9,10]. However, the evidence for mechanistic coordination among these processes is uneven. A direct physical linkage between nuclear pore complexes and bundled microtubules during nuclear elongation has been demonstrated [23], whereas the molecular mechanisms coordinating cyst organization, spermatid elongation, and the onset of individualization remain less well defined. Thus, the major unresolved question is not which structures participate in spermatid elongation, but how their activities are coordinated at the molecular level.

## 3. Mitochondrial Remodeling as a Structural–Metabolic Axis

**Figure 2:**
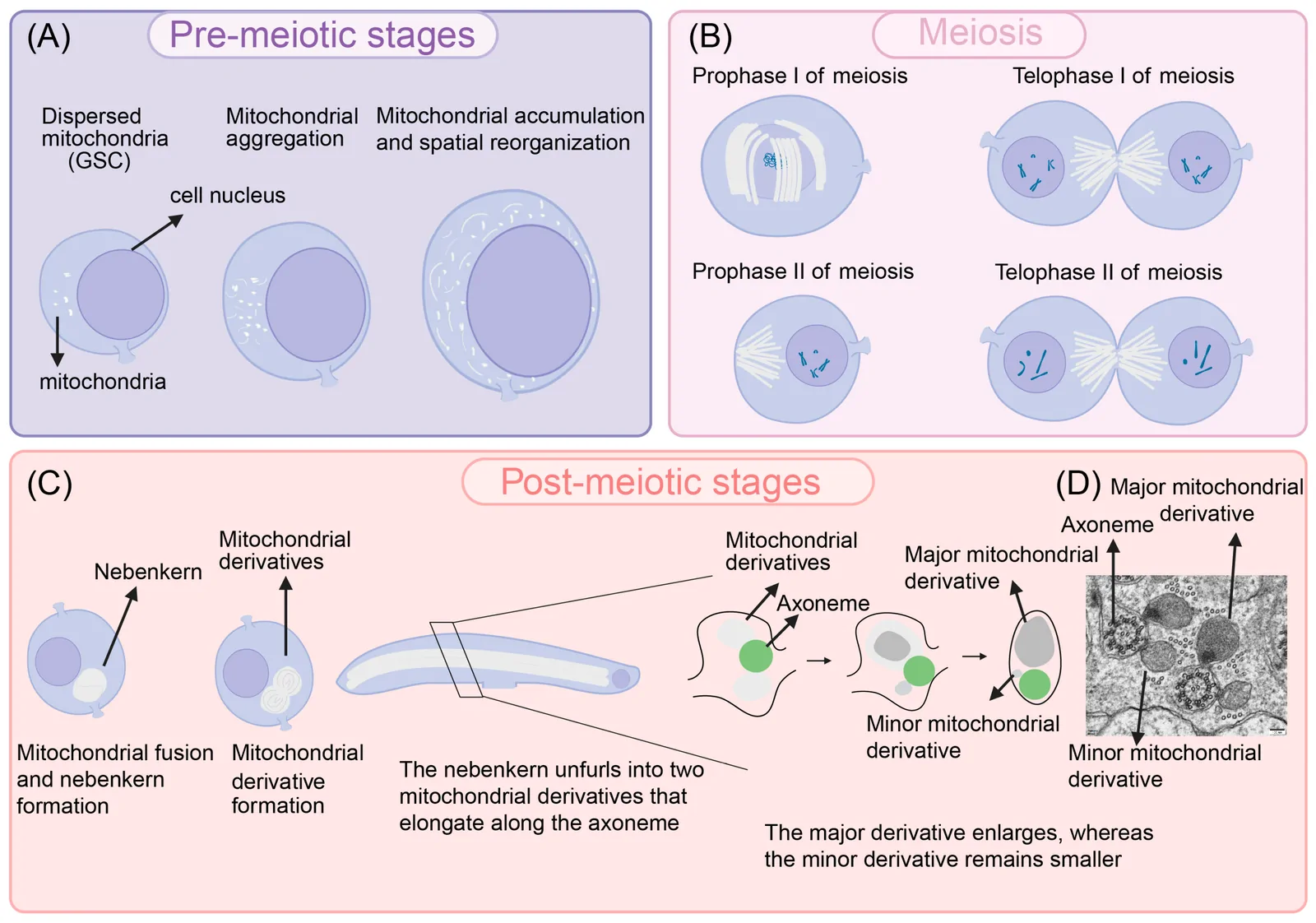
Dynamic mitochondrial remodeling during *Drosophila* spermiogenesis. (**A**) During spermatocyte development, mitochondria undergo redistribution, accumulation, and spatial reorganization. (**B**) During meiosis, mitochondria are partitioned among the four haploid round spermatids. (**C**) Following meiosis, mitochondria fuse to form the onion-stage nebenkern. The two intertwined mitochondrial subunits subsequently unfurl to generate the major and minor mitochondrial derivatives, which elongate in parallel with the axoneme. During late spermiogenesis, the major derivative accumulates paracrystalline material, whereas the minor derivative progressively decreases in cross-sectional area. (**D**) Original transmission electron micrograph acquired by the authors, showing the axoneme and associated mitochondrial derivatives in an elongating spermatid. The microscopy image shown in (**D**) is original and was generated by the authors; it was not reproduced or adapted from a previously published source. Scale bar: 100 nm.

*Drosophila* spermatogenesis is a classical model for studying cell polarity establishment, organelle remodeling, and cellular morphogenesis. As a key stage of spermiogenesis, spermatid elongation is accompanied by dynamic mitochondrial remodeling throughout late germ cell differentiation and represents one of the major factors influencing sperm tail elongation, energy metabolism, and male fertility. Before meiosis, mitochondria in spermatocytes gradually transition from a dispersed distribution to a more aggregated state, accompanied by quantitative amplification and spatial reorganization, which lays a sufficient organelle foundation for subsequent cell division and differentiation (Figure 2A). During meiosis, mitochondria are equally partitioned into daughter haploid round spermatids via cytokinesis, ensuring that nascent cells obtain the basic structural and energy supply required for further development (Figure 2B). Following meiosis, mitochondria in round spermatids fuse extensively and assemble into the nebenkern, a distinctive structure formed by two closely associated mitochondrial masses (Figure 2C) [36,37]. Its internal organization is highly specialized, with abundant cristae and respiratory-chain components that support the metabolic demands of later spermatid elongation [38,39,40]. Proper formation of the nebenkern is therefore a key step in mitochondrial remodeling. Disruption of mitochondrial fusion leaves mitochondria dispersed and is associated with abnormal spermatid development, defective elongation, and reduced male fertility [41,42,43]. Once assembled, the nebenkern provides the structural starting point from which the major and minor mitochondrial derivatives subsequently unfurl and extend along the developing axoneme.

Recent studies have identified metabolic regulators that directly affect mitochondrial derivative maintenance during spermatid elongation. ND-42, a mitochondrial complex I component, is required for the maintenance of mitochondrial membrane potential and mitochondrial derivatives, and its depletion leads to defective spermatid elongation [12]. More recently, Vha68-3 was shown to contribute to spermatid elongation by maintaining mitochondrial metabolic homeostasis, with Vha68-3 deficiency disrupting mitochondrial function and pyruvate metabolism in elongated spermatids [14]. These findings extend the mitochondrial layer beyond organelle morphology and demonstrate that mitochondrial respiratory and metabolic functions contribute directly to the maintenance of elongating spermatids [12,14].

After nebenkern assembly, mitochondria enter a directional elongation phase and extend in coordination with the axoneme, together contributing to the formation of the approximately 1.8 mm long sperm tail. This process provides both structural support and an important basis for sperm morphogenesis [44]. At the onset of spermatid elongation, the two intertwined mitochondrial compartments of the nebenkern unfurl to form the major and minor mitochondrial derivatives. Both derivatives elongate alongside the axoneme. During late spermiogenesis, the major derivative accumulates paracrystalline material, whereas the minor derivative remains smaller (Figure 2D). These two derivatives are positioned on both sides of the newly assembled “9+2” axonemal microtubule structure, forming an axoneme–mitochondrial derivative core architecture. Directional mitochondrial elongation is closely associated with the cytoplasmic microtubule array surrounding the mitochondrial derivatives. In elongating *Drosophila* spermatids, Milton accumulates on mitochondria near the growing tail tip and is required for local microtubule sliding; disruption of Milton or its associated protein dMiro causes marked spermatid elongation defects [11]. The dMiro–Milton complex links mitochondria to kinesin-dependent microtubule processes and therefore provides a candidate molecular interface between mitochondrial remodeling and the cytoskeleton [43]. Several additional microtubule-associated factors have also been implicated in this process. Nebbish and Fascetto contribute to microtubule crosslinking, whereas Khc73 and the kinesin-13 family member Klp59D participate in the organization or dynamics of the microtubule array during spermatid elongation [43]. Together, these findings provide experimental support for coupling between mitochondrial derivatives and the surrounding cytoplasmic microtubule network. However, the precise motor-dependent mechanism of force transmission and whether this machinery forms a direct molecular linkage to the axoneme itself remain unresolved. During this process, mitochondrial membranes undergo continuous lipid synthesis and expansion, allowing the mitochondrial derivatives to maintain elongated tubular structures without further division or proliferation. They extend synchronously and directionally with the axoneme and eventually run along almost the entire sperm tail, becoming one of its most prominent structural components. Unlike mammalian sperm mitochondria, which are mainly associated with energy production in the midpiece, *Drosophila* sperm mitochondria are not merely energy-supplying organelles but directly participate in cellular morphogenesis. Their extension is highly coordinated with axoneme assembly in both direction and timing. When mitochondrial extension is disrupted, sperm tails may become shortened, bent, or structurally abnormal, ultimately leading to defective flagellar formation [45,46,47]. This stage of mitochondrial remodeling represents the transition from a spherical nebenkern to elongated tubular mitochondrial derivatives, thereby contributing to sperm tail rigidity while also providing sustained metabolic support for later sperm maturation.

Mitochondrial quality-control mechanisms may also contribute to the maintenance of mitochondrial structure and function during spermatid elongation [48,49,50,51]. PINK1 and Parkin are established regulators of mitochondrial integrity and dynamics, including in *Drosophila* models [52,53], but direct evidence that the canonical PINK1–Parkin pathway regulates mitochondrial remodeling during spermatid elongation or individualization remains limited. By contrast, Drp1-mediated mitochondrial remodeling is supported by direct genetic evidence in *Drosophila* spermatogenesis [54]. Loss of Drp1 causes abnormal mitochondrial organization in spermatocytes and defective unfurling of mitochondrial derivatives in early elongating spermatids [54]. More broadly, Drp1 is an important regulator of mitochondrial structure and quality surveillance [55]. The PINK1–Parkin pathway is well established as a mitochondrial quality-control mechanism in other cellular contexts, where mitochondrial damage can promote PINK1-dependent recruitment and activation of Parkin, followed by ubiquitination of mitochondrial proteins and engagement of downstream autophagic machinery [56,57,58]. Whether this canonical sequence operates during *Drosophila* spermatid elongation or individualization has not been directly demonstrated. Although interactions between Drp1 and PINK1–Parkin have been reported in other cellular contexts, whether such interactions occur during *Drosophila* spermatid elongation or individualization is currently unknown.

During *Drosophila* spermatid elongation, mitochondria are not merely dynamically remodeled organelles; they may represent a central structural–metabolic hub that integrates structural support, energy supply, polarity establishment, and signaling-related functions [59,60,61]. Classic ultrastructural work by Tokuyasu established the close spatial relationship between the axoneme and mitochondrial derivatives during spermatid elongation [62]. As core structural elements of the sperm tail, mitochondrial derivatives contribute to the rigidity and morphological stability of the extremely long sperm tail and help maintain its elongated architecture. This structural organization provides an important basis for the later acquisition of sperm motility. In addition, mitochondria generate ATP through oxidative phosphorylation, supporting energy-consuming processes such as axoneme assembly, microtubule-based movement, membrane expansion, and nuclear condensation. Mitochondrial derivatives are associated with cytoplasmic microtubule arrays involving Milton–dMiro and other microtubule-regulatory factors, but how this local machinery is mechanically integrated with axonemal elongation remains unclear. Moreover, mitochondrial functional status may affect late spermatid differentiation through energy metabolism, redox state, or proteostasis. However, direct signaling from mitochondrial state to translational regulation or cytoskeletal remodeling has not yet been established in *Drosophila* spermatids, and these cross-layer relationships remain hypothetical. Mitochondrial defects can therefore affect several aspects of spermatid development beyond energy production. Abnormal mitochondrial remodeling is often accompanied by defects in axoneme organization, cell polarity, tail elongation, and sperm morphology, ultimately compromising male fertility [63,64,65,66]. Together with the extensive contribution of mitochondrial derivatives to the sperm tail, these observations support both structural and metabolic roles for mitochondria during *Drosophila* spermiogenesis, while their direct regulatory links to the cytoskeletal or translational machinery remain to be established.

## 4. Cytoskeleton and Individualization

**Figure 3:**
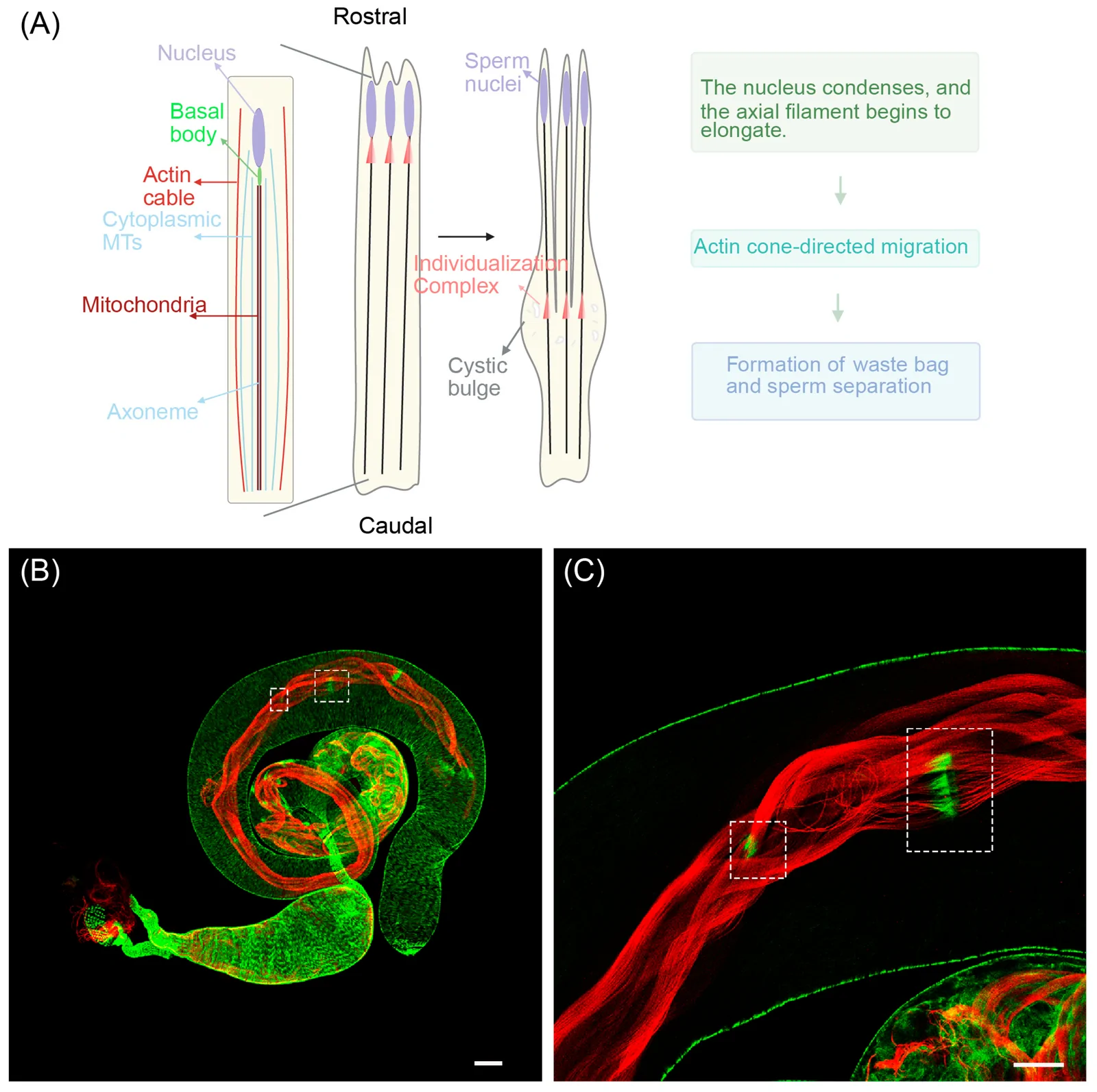
Assembly, migration, and cytoplasmic clearance of the individualization complex during *Drosophila* spermatogenesis. (**A**) Schematic illustration of the formation and migration of the individualization complex during late spermatid elongation in *Drosophila*. The individualization complex assembles near the sperm head region and subsequently progresses directionally along the sperm bundle from the head toward the tail. During migration, it progressively compresses and removes excess cytoplasm and abnormal organelles, forming the cystic bulge and the terminal waste bag, and ultimately separates the interconnected spermatids into individual mature sperm. (**B**) Whole-mount fluorescence image acquired by the authors, showing the spatial distribution of cytoskeleton-associated structures during the individualization stage of *Drosophila* sperm bundles. Phalloidin labels F-actin and is used to visualize the actin cones and the individualization complex (green), whereas AXO49 labels polyglycylated axonemal tubulin (red). (**C**) Enlarged view of the dashed-box region in (**B**), showing more clearly the spatial relationship between the individualization complex and the sperm bundle, as well as the local organization of actin cones during their migration along the sperm bundle. Together, these panels illustrate the important role of the individualization complex as a key execution machinery during the terminal stage of *Drosophila* sperm maturation, mediating cytoplasmic clearance, sperm separation, and morphological maturation. The microscopy images in (**B**,**C**) are original images generated by the authors and were not reproduced or adapted from previously published sources. Scale bar: 50 μm (**B**); 25 μm (**C**).

Spermatid individualization in *Drosophila* is a critical terminal step of sperm maturation and is executed by the individualization complex, a cytoskeleton-based structure that drives sperm separation and removal of redundant cellular materials. The molecular composition and spatial organization of this complex are highly specialized [67,68,69,70,71,72]. The individualization complex is built around actin cones, funnel-shaped F-actin-rich structures that assemble near the sperm head region, migrate along the sperm bundle, and drive cytoplasmic clearance, cystic bulge formation, waste-bag formation, and final separation of interconnected spermatids (Figure 3A). In addition to the actin cytoskeleton, the complex incorporates multiple regulatory and cytoskeleton-associated proteins, including actin-polymerizing factors such as the Arp2/3 complex, actin crosslinking proteins, myosin motor proteins that drive cellular movement, and regulatory factors involved in ubiquitination and membrane remodeling. Together, these components form a stable but dynamically active functional complex. The individualization complex operates at the level of the 64-cell spermatid cyst. Each spermatid contains one actin cone, and all actin cones within the cyst remain synchronously aligned and function cooperatively, forming an ordered cytoskeletal array [73,74,75,76]. Its assembly strictly depends on polarity cues established during late spermatid elongation. Only spermatids that have completed axoneme extension and mitochondrial derivative elongation are competent to initiate the ordered assembly of the individualization complex. Structural defects in this complex can directly arrest individualization, preventing separation of the 64 interconnected spermatids and ultimately leading to the formation of nonfunctional syncytial sperm bundles.

Molecular studies have identified several regulators acting at distinct steps of individualization. Myosin VI is concentrated in the leading region of the actin cone and stabilizes the dense actin network required for efficient cytoplasmic exclusion, indicating that its major role is actin-network stabilization rather than simply cargo transport [8]. LKB1 is required in the germline for formation of the polarized actin cones that initiate individualization [74], whereas Pif1A is required for the coordinated movement of these cones along the spermatid bundle [75]. More recently, Mob4 was shown to regulate axonemal organization and spermatid individualization as a component of the conserved STRIPAK complex; depletion of Mob4 or other STRIPAK components disrupts cytoskeletal organization and causes male sterility [68]. A recent study further showed that the C-terminal ZZ domain of the RNA-binding protein Orb2 is required for spermatid individualization; its deletion disrupts the localization of ORB, IMP, and SOTI and abolishes formation of the SOTI-dependent caspase gradient [72]. ζTrypsin has also recently been identified as a factor required for spermatid elongation and individualization [71], although its direct molecular targets remain unresolved. These findings reveal that individualization depends on molecularly distinct mechanisms controlling actin-cone assembly, stabilization and movement, axonemal organization, and additional regulatory processes associated with cytoplasmic clearance.

The dynamic migration of the individualization complex is the central process of spermatid individualization. This process depends on cytoskeletal regulation and motor protein activity to achieve unidirectional and synchronized movement from the sperm head toward the tail in a precise and orderly manner (Figure 3B,C) [77]. After assembly of the complex, myosin motor proteins hydrolyze ATP to generate force, driving actin cones to move along the longitudinal axis of the spermatid bundle from the posterior nuclear region toward the distal tail. During this migration, the 64 actin cones within the same cyst move synchronously, without obvious lagging or premature advancement, thereby ensuring coordinated individualization. The axoneme and mitochondrial derivatives provide the longitudinal structural context for this process, whereas actin cones and their associated regulatory factors act as the direct execution machinery for cytoplasmic removal and sperm separation. How individualization-complex migration is regulated by broader metabolic or stress-response pathways remains less well established, and direct evidence linking these pathways to actin-cone dynamics is currently limited. Proper actin turnover and motor activity are required for the coordinated movement of the individualization complex; when either process is disturbed, the complex may stall, break apart, or lose its normal direction of travel. Throughout this movement, the actin cones retain their characteristic funnel-like shape and remain closely apposed to the spermatid membrane. Their passage along the bundle pushes cytoplasmic material toward the distal end, while progressively separating neighboring spermatids and removing the intercellular connections that maintain the syncytium. This process converts the shared spermatid bundle into individually separated sperm cells.

As the individualization complex moves along the spermatid bundle, it removes much of the cytoplasmic material that is no longer required for sperm maturation. The advancing actin cones physically displace excess cytoplasm, residual organelle material, and discarded proteins toward the distal end of the cyst, where these components accumulate in the waste bag [78]. Cytoplasmic removal is accompanied by protein-degradation pathways involving ubiquitin–proteasome components and other degradation-related factors associated with individualization. Together, these mechanical and degradative processes help eliminate unnecessary cellular material while the spermatids acquire their final streamlined morphology. This clearance mechanism is highly selective: functional structures such as the axoneme and mitochondrial derivatives are retained, whereas nonessential cytoplasmic components are precisely removed, greatly reducing spermatid volume and allowing sperm to acquire a streamlined mature morphology [79,80]. In addition, the plasma membrane is remodeled in coordination with complex migration, allowing membrane reorganization to occur simultaneously with cytoplasmic clearance and enclosing each sperm within an intact membrane structure. If cytoplasmic clearance is impaired, excess cytoplasm cannot be properly removed, resulting in swollen or disorganized sperm morphology, loss of motility, and ultimately male infertility. Thus, this clearance mechanism is essential for the acquisition of functional mature sperm.

Together, these studies establish the actin-based individualization complex as the principal execution machinery for cytoplasmic clearance and spermatid separation. Genetic and imaging evidence strongly supports the roles of actin-cone organization and migration in this process, whereas the upstream mechanisms that coordinate individualization with mitochondrial status, energy metabolism, or other regulatory layers remain less well defined. Thus, current evidence is strongest for the local cytoskeletal machinery, while broader cross-layer regulation requires further mechanistic validation.

## 5. Delayed Translation and Developmental Timing Under Transcriptional Silencing

**Figure 4:**
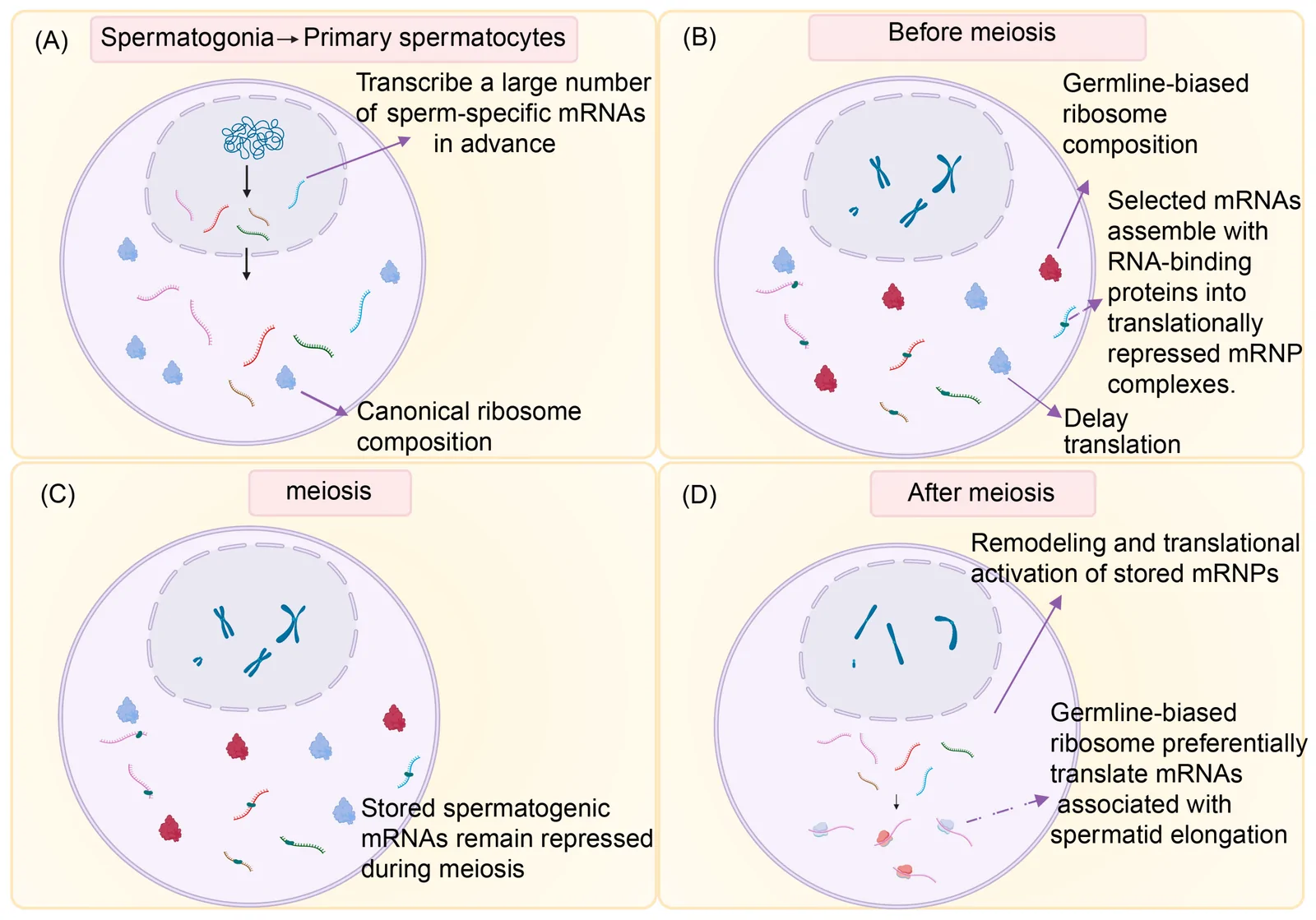
Delayed translation and potential ribosome-related regulation during *Drosophila* spermatogenesis. (**A**) From the spermatogonial stage to the primary spermatocyte stage, cells transcribe large amounts of spermatogenesis-related mRNAs in advance, providing the template basis for protein expression during post-meiotic spermatid elongation and maturation. (**B**) During the premeiotic stage, a subset of spermatogenesis-related mRNAs associates with translational repressors to form translationally repressed mRNP complexes, thereby preventing premature protein expression. At the same time, germ cells may exhibit stage-specific ribosomes or changes in ribosome composition, providing a potential basis for subsequent selective translation. (**C**) During meiosis, the stored mRNAs largely remain in a translationally repressed state, delaying the expression of spermatogenesis-related proteins until the appropriate developmental stage and thereby ensuring temporal coordination between meiosis and subsequent spermatid differentiation. (**D**) After meiosis, as round spermatids enter the elongation stage, some mRNP complexes are released from translational repression, and stored mRNAs are re-recruited to the translational machinery to produce proteins associated with spermatid elongation, including those involved in axoneme assembly, mitochondrial remodeling, cytoskeletal regulation, and individualization. This figure summarizes the experimentally supported delayed-translation program in *Drosophila* spermatogenesis, characterized by “early transcription–translational repression–post-meiotic release–temporally regulated translation.” Ribosome compositional specificity is shown separately as a proposed mechanism that may contribute to selective protein expression during spermatid elongation but remains to be directly demonstrated. Light-blue ribosome symbols indicate canonical ribosome composition, whereas dark-red ribosome symbols indicate germline-biased ribosome composition. Differently colored wavy lines represent spermatogenesis-related mRNAs and are used only for visual distinction. In panels B and C, the small symbols associated with selected mRNAs represent RNA-binding proteins within translationally repressed mRNP complexes. Solid arrows indicate the established delayed-translation sequence, whereas dashed arrows indicate proposed ribosome-related regulatory relationships.

Spermatid elongation in *Drosophila* depends extensively on post-transcriptional regulation that allows protein production to be separated temporally from mRNA synthesis. During late spermiogenesis, progressive chromatin compaction restricts transcriptional capacity, while many genes required for post-meiotic differentiation are transcribed earlier in primary spermatocytes and their mRNAs are stored in a translationally repressed state until the corresponding proteins are required [81,82,83,84]. This transcription–translation delay, summarized in Figure 4A–C, has been demonstrated for several well-characterized spermatogenesis genes. For example, *don juan* mRNA is transcribed in primary spermatocytes but remains translationally repressed through meiosis and early spermatid development before being translated during spermiogenesis [84]. Similarly, the expression of protamine B and Mst77F is controlled by transcription in spermatocytes followed by translational repression and later protein accumulation in elongating spermatids [83]. These studies establish delayed translation as an important mechanism for matching protein production to specific stages of spermatid differentiation. Genetic analyses of germline translation factors, including eIF4E-family proteins and eIF4G/eIF4G2, further support the requirement for regulated translation during meiosis and spermatid differentiation [18,19]. More recent studies have extended this regulatory framework to the tRNA modification machinery. Mettl1-dependent m7G modification stabilizes a subset of tRNAs, and loss of Mettl1 reduces the abundance of these tRNAs and causes codon-specific ribosome stalling [85]. Ribosome profiling further showed reduced translational efficiency of genes involved in elongated spermatid formation and sperm stability in Mettl1-deficient testes [85]. These findings provide a direct molecular link between tRNA modification, translational efficiency, and spermatid differentiation.

Genetic studies of germline-specific ribosomal protein paralogs and translation factors further suggest that the translational machinery itself may be developmentally specialized during spermatogenesis [17,18,19,86,87]. However, whether specific ribosomal subtypes directly and selectively translate mRNAs associated with spermatid elongation remains to be demonstrated by ribosome profiling (Ribo-seq) or interactome analyses of specialized ribosomes [86,87]. Traditionally, ribosomes were considered relatively uniform cellular machines. However, accumulating evidence suggests that ribosome composition, rRNA modifications, and structural features can vary in a cell type- or developmental stage-specific manner, giving rise to ribosome heterogeneity (Figure 4D). In *Drosophila* spermatogenesis, such ribosome heterogeneity may contribute to selective translational regulation during late germ cell differentiation. During spermatid elongation, germ cell-specific ribosomal protein paralogs may replace conventional ribosomal components and thereby alter ribosomal properties related to mRNA recognition or translational efficiency. In this way, ribosome heterogeneity could help optimize translation of mRNAs involved in axoneme assembly, mitochondrial derivative elongation, and cytoskeletal remodeling, while also ensuring the efficient use of translational resources under transcriptionally repressed conditions. If the expression of ribosomal protein paralogs is disrupted, the assembly or function of specialized ribosomes may be impaired, potentially resulting in insufficient translation of proteins required for sperm development, abnormal sperm morphology, incomplete elongation, and reduced male fertility. Evidence for ribosome heterogeneity in the *Drosophila* germline is emerging, but direct demonstration that distinct ribosome populations selectively translate spermatid-elongation transcripts remains limited. Therefore, specialized ribosomes are discussed here as a potential additional layer of translational specificity rather than as an established component of the elongation program.

Overall, delayed translation is supported by stage-specific regulation of stored germline mRNAs and by genetic evidence for multiple translation factors. In contrast, ribosomal paralog-specific phenotypes establish a requirement for particular ribosomal components but do not yet demonstrate selective translation of a defined set of elongation-related mRNAs. Resolving this distinction will require stage-resolved Ribo-seq, ideally combined with direct characterization of ribosome-associated transcripts. Figure 4 therefore summarizes the established delayed-translation program while separately indicating ribosome compositional specificity as a potential, but not yet fully demonstrated, mechanism of selective translation.

## 6. An Integrated Regulatory Model

The major cellular and molecular processes involved in *Drosophila* spermatid elongation and individualization, together with representative regulatory factors, experimentally supported functions, and current limitations, are summarized in Table 1.

Based on the evidence discussed above, this review proposes a working model in which *Drosophila* spermatid elongation is coordinated by three interconnected regulatory layers: mitochondrial remodeling, cytoskeletal execution, and post-transcriptional translational control. In this model, mitochondrial remodeling provides a structural–metabolic layer through nebenkern formation, mitochondrial derivative elongation, and energy metabolism; the cytoskeleton provides the execution layer through axoneme assembly, microtubule organization, actin cone migration, and individualization; and post-transcriptional regulation provides the temporal layer by controlling the staged translation of proteins required for elongation and maturation.

At the molecular level, each layer is supported by experimentally defined regulatory mechanisms. Mitochondrial derivative maintenance involves mitochondrial respiratory and metabolic regulation, including ND-42- and Vha68-3-dependent processes [12,14]. Cytoskeletal execution involves regulators of actin-cone stabilization, assembly, and movement, as well as axonemal organization, including Myosin VI, LKB1, Pif1A, and Mob4 [8,68,74,75]. Post-transcriptional control involves delayed translation, translation-initiation factors, and tRNA modification-dependent regulation of translational efficiency [18,19,83,84,85]. These experimentally supported within-layer mechanisms should nevertheless be distinguished from the still incompletely resolved molecular interactions between the three layers.

The evidence supporting the three layers is asymmetric. Mitochondrial morphogenesis, axoneme/actin-based remodeling, and delayed translational control are each supported by direct genetic and cell-biological observations, whereas evidence for communication between these layers is largely indirect. Spatial association, overlapping mutant phenotypes, and developmental synchrony support functional coordination, but do not by themselves establish direct molecular coupling or pathway order. TOR signaling may provide an upstream link between nutrient status and the three regulatory processes discussed above. TORC1 responds to nutrient and growth signals and regulates protein synthesis through downstream effectors including 4E-BP and S6K [88,89,90]. It also affects mitochondrial metabolism, linking nutrient availability with translational capacity and cellular energy metabolism [91]. In addition, TORC2 has been associated with actin-cytoskeletal regulation in several cellular systems [92]. In the *Drosophila* testis, TORC1 activity has been detected in germline stem cells and early germ cells, and reduced *Tor* activity impairs germ-cell differentiation [93]. Whether TOR has a similar function during post-meiotic spermatid elongation remains unknown. The available evidence therefore supports a role for TOR signaling in male germline development and provides a basis for considering it as a possible upstream regulator of metabolism, translation, and cytoskeletal organization. However, a direct integrative role during *Drosophila* spermatid elongation remains to be demonstrated.

At the same time, this model should be interpreted as a working hypothesis rather than a fully established causal pathway. The direct molecular links between mitochondrial derivatives, the axoneme, actin-based individualization, and translational regulation remain incompletely defined. Future studies should therefore test the direction and hierarchy of these interactions using genetic interaction analysis, live imaging, ultrastructural time courses, ribosome profiling, proteomics, and comparative single-cell approaches. Such work will help determine whether mitochondrial remodeling, cytoskeletal dynamics, and translational control form a true regulatory network or represent parallel processes that converge during spermatid morphogenesis.

**Figure 5:**
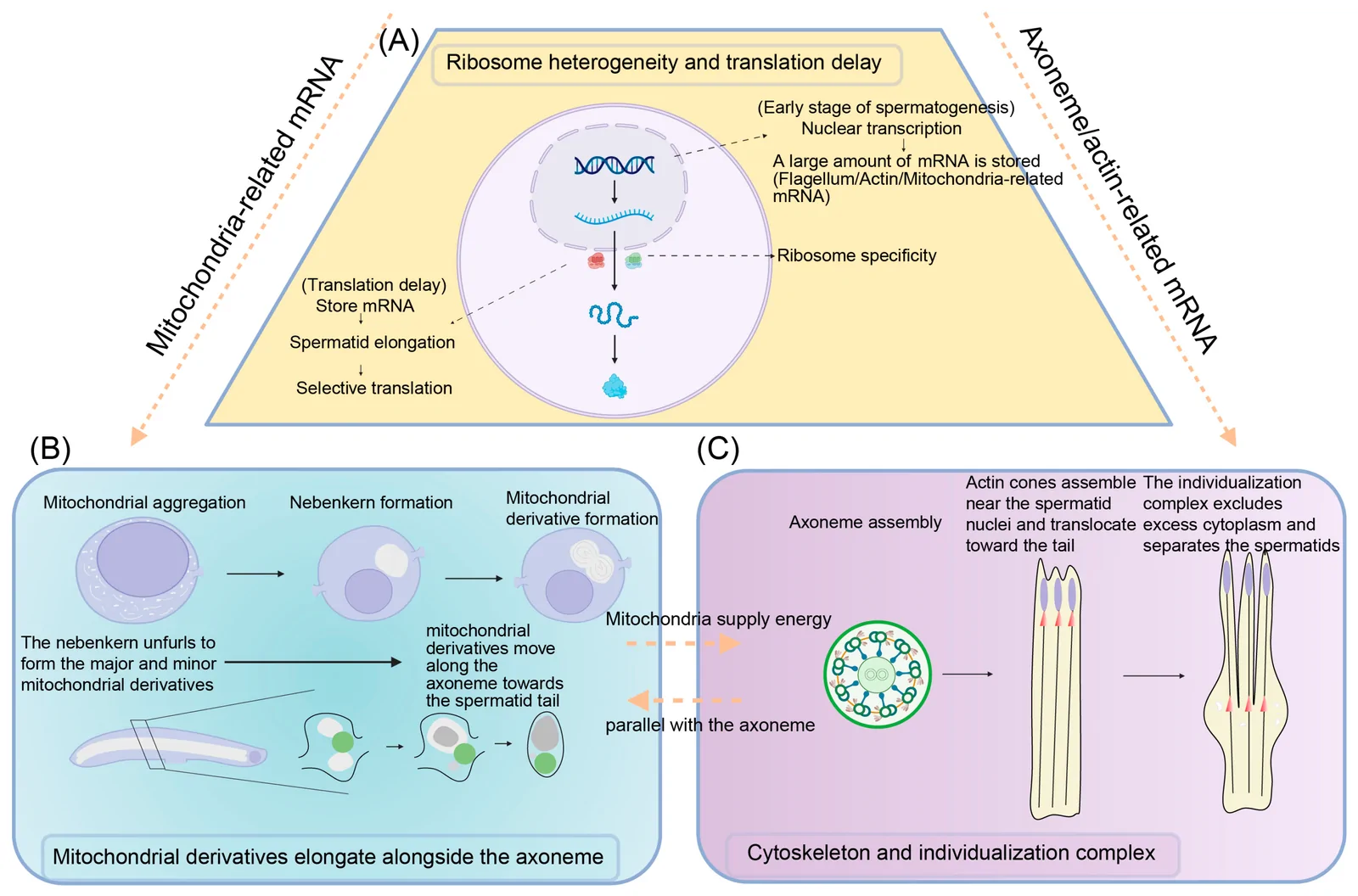
Evidence-based working model linking mitochondrial remodeling, cytoskeletal dynamics, and translational control during *Drosophila* spermatid elongation. (**A**) Post-transcriptional/translational regulatory layer. During early spermatogenesis, the nucleus remains transcriptionally active and can transcribe and store large amounts of mRNAs related to mitochondrial remodeling, axoneme assembly, and cytoskeletal regulation. As spermatids enter the post-meiotic differentiation stage, chromatin gradually condenses and transcriptional activity declines. Stored mRNAs are then expressed in a temporally controlled manner through delayed translation and potential ribosome compositional specificity, thereby providing structural proteins and regulatory factors required for spermatid elongation. (**B**) Mitochondrial remodeling layer. After meiosis, mitochondria further fuse from an aggregated state to form the nebenkern, which subsequently undergoes reorganization and differentiates into mitochondrial derivatives that extend along the axoneme. These mitochondrial derivatives not only provide structural support for sperm tail elongation, but also supply metabolic support through energy metabolism for axoneme assembly, membrane extension, and the individualization process. (**C**) Cytoskeletal and individualization execution layer. Axoneme assembly constitutes the core scaffold for sperm tail elongation. Subsequently, actin cones form the individualization complex and migrate along the sperm bundle from the head toward the tail, driving the removal of excess cytoplasm and abnormal organelles and ultimately achieving spermatid separation. This model summarizes the potential coupling relationships among post-transcriptional/translational regulation, dynamic mitochondrial remodeling, and cytoskeletal execution during *Drosophila* spermatid elongation. Solid arrows indicate experimentally supported developmental relationships, whereas dashed arrows indicate proposed or incompletely resolved cross-layer interactions. Background colors distinguish the three regulatory layers; black solid arrows indicate developmental or structural progression, orange dashed arrows indicate proposed cross-layer relationships, and colored ribosome symbols indicate ribosome compositional heterogeneity. Accordingly, the figure should be interpreted as an evidence-based working model rather than an established linear signaling pathway.

In summary, the mitochondria–cytoskeleton–translation model provides a practical framework for organizing current knowledge of *Drosophila* spermatid elongation. Its main purpose is not to claim that one pathway dominates the entire process, but to emphasize that sperm-tail elongation and maturation require coordinated structural, metabolic, and post-transcriptional regulation. This framework may also guide future comparisons between *Drosophila*, mammalian spermatogenesis, and human male infertility-associated genes. The proposed coupling among mitochondrial remodeling, cytoskeletal execution, and translational control is illustrated in Figure 5.

## 7. Comparative Conservation of Spermatogenesis Between *Drosophila* and Humans

Spermatogenesis in *Drosophila* and mammals shares several conserved cellular processes, including meiosis, axoneme formation, chromatin compaction, mitochondrial regulation, cytoplasmic remodeling, and post-transcriptional control. However, conservation of these general processes should not be interpreted as conservation of their cellular architecture or execution. Three differences are particularly relevant to the present review: the distribution and structural contribution of mitochondria, the organization of the sperm tail, and the mechanism of cytoplasmic removal during terminal differentiation. *Drosophila* spermatids develop synchronously within a 64-cell cyst and undergo extreme tail elongation, whereas mammalian spermatids differentiate within the seminiferous epithelium in close association with Sertoli cells. These distinctions place important limits on direct mechanistic extrapolation between the two systems [94].

The core axonemal organization is one of the more conserved features of sperm development, with both *Drosophila* and mammalian sperm containing the canonical “9+2” microtubule arrangement and dynein-associated machinery. The surrounding tail architecture, however, differs substantially. The mature *Drosophila* axoneme is associated with an additional set of accessory microtubules, producing the characteristic “9+9+2” organization, whereas mammalian sperm tails contain outer dense fibers and a fibrous sheath and are compartmentalized into midpiece, principal-piece, and end-piece regions [94]. These differences indicate that conservation of the axonemal core does not imply conservation of the complete flagellar architecture. In humans, defects in axonemal dynein assembly, CFAP-family proteins, and other flagellar structural factors are associated with asthenozoospermia, immotile sperm phenotypes, and multiple morphological abnormalities of the sperm flagella (MMAF) [95,96,97,98,99,100]. These findings provide a clinically relevant framework for interpreting *Drosophila* mutants with defective axoneme assembly, abnormal sperm-tail morphology, or reduced male fertility.

Mitochondrial organization represents an even more pronounced structural difference between the two systems. In mammalian sperm, mitochondria are confined to the midpiece, where they form a mitochondrial sheath around the proximal flagellar structures. In *Drosophila*, by contrast, the nebenkern gives rise to the major and minor mitochondrial derivatives, which extend along almost the entire length of the exceptionally long sperm tail and contribute directly to tail architecture [94,101,102,103]. Thus, although mitochondrial integrity, membrane potential, and energy metabolism are important for sperm development and function in both systems, the spatial organization and structural roles of sperm mitochondria are fundamentally different. Mitochondrial phenotypes observed in *Drosophila* can therefore inform general principles of mitochondrial regulation, but their morphological consequences should not be assumed to correspond directly to those in mammalian sperm. Cytoplasmic clearance is another process in which similar developmental outcomes are achieved through distinct cellular mechanisms. In *Drosophila*, the 64 actin cones of the individualization complex migrate synchronously along the elongated spermatid bundle, removing excess cytoplasm and membrane components into a cystic bulge that ultimately forms the waste bag [8,9,10,67,68,69,70,71,72,73,74,75,76,77,78,79,80]. Mammalian spermatids do not undergo an equivalent cyst-wide actin-cone migration. Instead, excess cytoplasm is segregated into residual bodies during spermiation, and much of this material is subsequently phagocytosed by Sertoli cells [94]. The *Drosophila* waste bag and the mammalian residual body therefore represent broadly analogous outcomes of cytoplasmic reduction, but the cellular machinery and somatic-cell involvement are distinct.

**Figure 6:**
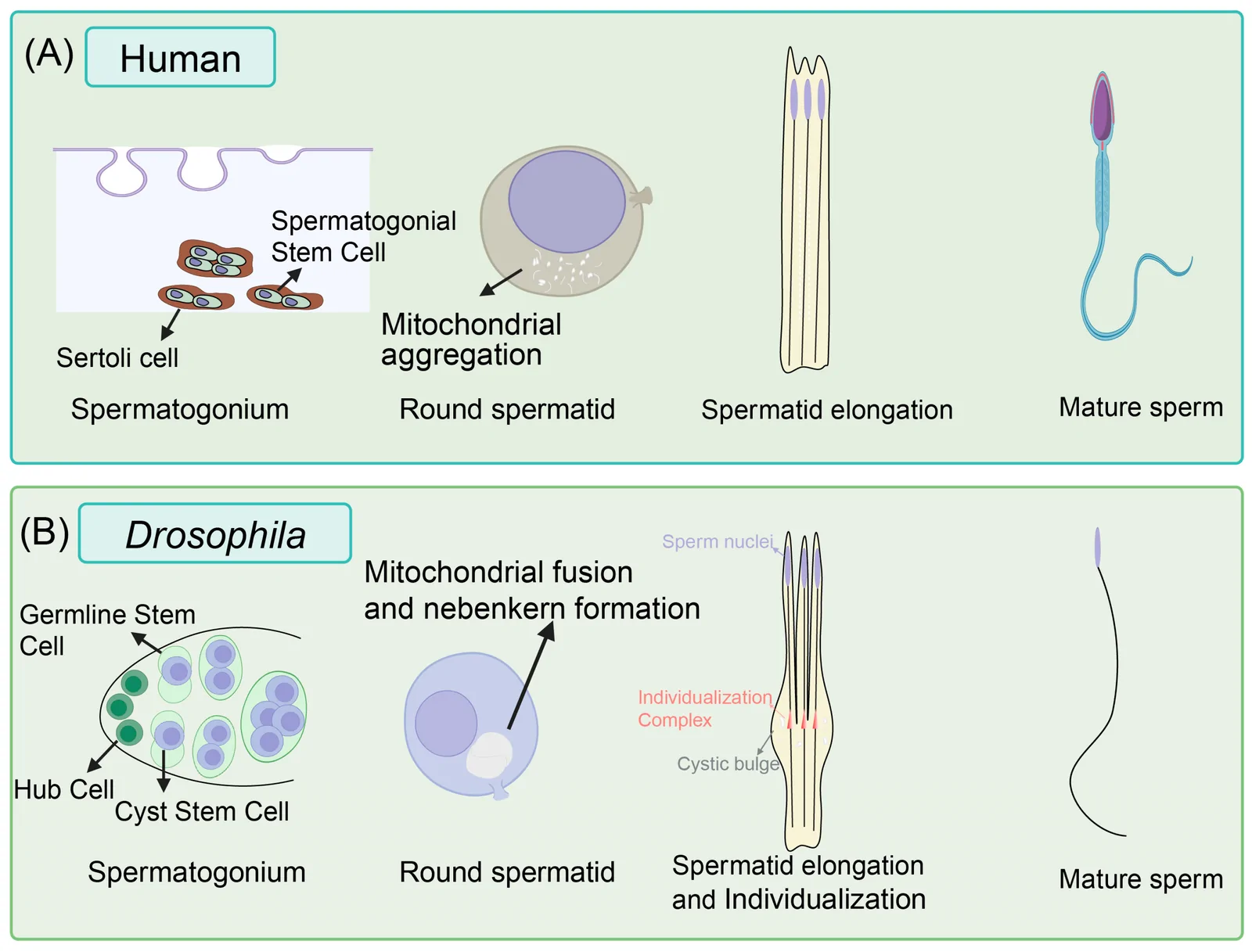
Comparison of key stages of spermatogenesis between humans and *Drosophila*. (**A**) Schematic illustration of human spermatogenesis. After spermatogonial proliferation, meiosis, and the formation of haploid spermatids, germ cells enter the stages of spermatid elongation and maturation. During this process, the nucleus gradually condenses and forms the sperm head, while mitochondria are mainly concentrated in the sperm midpiece to provide energy for flagellar motility, ultimately producing mature sperm with a head, midpiece, and tail. (**B**) Schematic illustration of *Drosophila* spermatogenesis. After mitotic amplification and meiosis, *Drosophila* spermatogonia give rise to round spermatids. Mitochondria subsequently fuse to form the nebenkern, which further differentiates into mitochondrial derivatives and extends along the axoneme, contributing to the structural formation of the extremely long sperm tail. After elongation is completed, the individualization complex migrates along the sperm bundle from the head toward the tail, removes excess cytoplasm, and forms the waste bag, ultimately separating the interconnected spermatids into individual mature sperm. This figure shows that human and *Drosophila* spermatogenesis share partial conservation in fundamental processes such as meiosis, axoneme formation, nuclear condensation, and mature sperm formation. However, the two systems differ markedly in mitochondrial distribution, tail architecture, and mechanisms of cytoplasmic clearance. Therefore, the *Drosophila* model can be used to investigate conserved cellular processes such as spermatid elongation and individualization, but the corresponding mechanisms should not be assumed to be directly equivalent to those in human spermatogenesis.

Late spermatogenesis in both *Drosophila* and mammals is also characterized by reduced transcriptional activity and increased dependence on post-transcriptional regulation. Stored mRNAs, RNA-binding proteins, delayed translation, poly(A)-tail regulation, and ribosome-associated mechanisms help to ensure that key proteins are produced at the appropriate developmental stages [104,105]. These similarities support the use of *Drosophila* to investigate general principles of translational timing during spermatid differentiation. Overall, the main value of the *Drosophila* model is not that it fully reproduces human spermatogenesis, but that it enables genetic and cellular dissection of conserved processes relevant to sperm morphogenesis and selected forms of human male infertility [106,107,108,109,110]. The comparison between human and *Drosophila* spermatogenesis should therefore be understood as a comparison of partially conserved cellular modules rather than a direct one-to-one correspondence between two identical developmental systems (Figure 6).

## 8. Conclusions

Spermatid elongation in *Drosophila* is supported by a substantial body of genetic and cell-biological work, although the strength of evidence is not uniform across the processes discussed in this review. The roles of mitochondrial morphogenesis, axoneme organization, actin-cone-mediated individualization, and delayed translation are supported by mutant analyses, imaging, and functional studies. The links among these processes are less certain. Mitochondrial derivatives and the axoneme elongate in parallel, and defects in one process are often accompanied by abnormalities in the other, but such observations do not establish a direct physical or regulatory connection. A similar distinction applies to ribosome-related regulation. Phenotypes caused by disruption of particular ribosomal paralogs support their requirement during spermatogenesis, but they do not yet show that distinct ribosome populations selectively translate a defined set of elongation-related mRNAs. We therefore regard the three-layer model as a framework for organizing current evidence rather than as an established causal pathway. Several of these uncertainties can now be addressed experimentally. Genetic interaction analyses and live imaging could help determine whether mitochondrial remodeling directly affects cytoskeletal organization or the timing of individualization. Ribo-seq at defined developmental stages, together with analysis of ribosome-associated transcripts, will be important for testing whether ribosome composition contributes to selective translation. Time-resolved ultrastructural and proteomic analyses may further clarify the order in which structural and molecular changes occur during elongation. Such work should help distinguish direct mechanistic coupling from parallel processes that converge during spermatid morphogenesis, and will also provide a firmer basis for assessing which features of this regulatory organization are conserved in mammalian spermatogenesis and male infertility.

## Figures and Tables

**Table 1:** Major cellular and molecular processes involved in *Drosophila* spermatid elongation and individualization.

Cellular or Molecular Process	Main Biological Process	Representative Factors or Structures	Evidence Status and Current Interpretation	Representative References
Developmental framework and cyst organization	Germline stem cell asymmetric division, spermatogonial amplification, cyst cell enclosure, meiosis, and formation of 64 interconnected round spermatids.	Germline stem cells, cyst stem cells, cyst cells, cytoplasmic bridges, spermatogonial cysts, primary spermatocytes, round spermatids.	Provides the spatially restricted developmental microenvironment and synchronizes germ-cell differentiation before spermatid elongation. Cyst polarity further establishes the spatial reference for subsequent nuclear positioning, axoneme extension, and sperm-bundle organization.	[1,2,3,24,25,26,27,28,29]
Axoneme and microtubule cytoskeleton	Basal body-derived axoneme assembly, microtubule elongation, dynein arm organization, and sperm-tail scaffold formation.	Basal body, “9+2”axoneme, peripheral doublet microtubules, central pair microtubules, dynein arms, TBCEL/mulet, Yuri gagarin, Wampa, roadblock, CFAP-related factors.	Forms the core scaffold for sperm-tail elongation and later motility. Defects in axonemal assembly or microtubule-associated factors can impair tail extension, flagellar structure, and male fertility.	[4,5,6,10,13,20,22,23,30,31,32,33,34,35]
Nuclear elongation and chromatin remodeling	Chromatin condensation, histone-to-protamine transition, nuclear shaping, and linkage between the nucleus and cytoskeletal structures.	Mst77F, protamines, Mst27D, nuclear pore complexes, bundled microtubules.	Drives the transition from round nuclei to highly condensed needle-like nuclei. Proper nuclear shaping requires both chromatin remodeling and mechanical linkage to cytoskeletal structures.	[7,23,27,81,83]
Mitochondrial remodeling	Mitochondrial aggregation, nebenkern formation, nebenkern unfolding, mitochondrial derivative elongation, and mitochondrial metabolic remodeling.	Nebenkern, major and minor mitochondrial derivatives, ND-42, Vha68-3, Marf, Opa1, Drp1, Milton, Spermitin, ATP synthase subunits, CG9920, M9.7-d.	Acts as a structural-metabolic layer supporting sperm-tail elongation. Mitochondrial derivatives contribute to tail architecture, mechanical stability, and energy metabolism. Their association with cytoplasmic microtubule arrays is supported by genetic and imaging evidence, whereas the molecular basis linking this machinery to axonemal elongation remains incompletely resolved.	[11,12,14,15,16,36,37,38,39,40,41,42,43,44,45,46,47,54,59,60,61,62]
Mitochondrial quality control	Maintenance of mitochondrial morphology, removal of damaged mitochondrial components, mitophagy-related regulation, and mitochondrial homeostasis.	Drp1, PINK1-Parkin pathway, ubiquitination machinery, Ref(2)P, autophagy-related factors, mitochondrial DNA removal mechanisms.	May help preserve mitochondrial function during spermatid differentiation and individualization. Direct genetic evidence supports Drp1-dependent mitochondrial remodeling during *Drosophila* spermatogenesis, whereas PINK1-Parkin is supported primarily by mitochondrial-integrity and fertility phenotypes; its direct role during spermatid elongation or individualization remains unresolved.	[48,49,50,51,52,53,54,55,56,57,58,66]
Actin cytoskeleton and individualization	Actin cone assembly, individualization complex migration, cytoplasmic clearance, membrane remodeling, waste-bag formation, and separation of interconnected spermatids.	Actin cones, individualization complex, Myosin VI, Arp2/3-related machinery, LKB1, Pif1A, Combover, Mob4, Orb2, ζTrypsin, cytoskeletal and motor proteins.	Executes the terminal maturation program by removing excess cytoplasm and separating 64 interconnected spermatids into individual mature sperm. Defects cause cytoplasmic retention, individualization failure, and male sterility.	[8,9,10,21,68,69,70,71,72,73,74,75,76,77,78,79,80]
Delayed translation and ribosome-related regulation	Premeiotic mRNA production and storage, mRNP formation, translational repression, post-meiotic release of stored mRNAs, and potential ribosome compositional specificity.	Stored mRNAs, mRNPs, RNA-binding proteins, eIF4E, eIF4E-3, eIF4E-5, eIF4G, eIF4G2, PAN2, microRNAs, RpS3, RpS25, eRpL22 paralogue-specific ribosomes, Mettl1, ValRS-m.	Provides temporally controlled protein expression under reduced transcriptional activity. Delayed translation is well supported, whereas the extent to which specialized ribosomes selectively translate elongation-related mRNAs requires additional Ribo-seq, ribosome profiling, and interactome evidence.	[9,17,18,19,81,82,83,84,85,86,87]

## Data Availability

Representative microscopy images generated by the authors are included in the article. The corresponding original images are available from the corresponding author upon reasonable request.

## Abbreviations

The following abbreviations are used in this manuscript:

ATP	adenosine triphosphate
AXO49	AXO 49 monoclonal antibody
CFAP	cilia- and flagella-associated protein
DAPI	4′,6-diamidino-2-phenylindole
Drp1	dynamin-related protein 1
eIF	eukaryotic initiation factor
F-actin	filamentous actin
GSCs	germline stem cells
MMAF	multiple morphological abnormalities of the flagella
mRNA	messenger RNA
mRNP	messenger ribonucleoprotein
ND-42	NADH dehydrogenase 42 kDa subunit
PINK1	PTEN-induced kinase 1
Ribo-seq	ribosome profiling sequencing
rRNA	ribosomal RNA
Vha68-3	vacuolar H+-ATPase 68 kDa subunit 3
